# Multi-session adaptation to audiovisual and sensorimotor biofeedback is heterogeneous among adolescents with cerebral palsy

**DOI:** 10.1371/journal.pone.0313617

**Published:** 2024-11-18

**Authors:** Alyssa M. Spomer, Benjamin C. Conner, Michael H. Schwartz, Zachary F. Lerner, Katherine M. Steele

**Affiliations:** 1 Department of Mechanical Engineering, University of Washington, Seattle, Washington, United States of America; 2 College of Medicine – Phoenix, University of Arizona, Phoenix, Arizona, United States of America; 3 James R. Gage Center for Gait & Motion Analysis, Gillette Children’s, Saint Paul, Minnesota, United States of America; 4 Department of Orthopedic Surgery, University of Minnesota, Minneapolis, Minnesota, United States of America; 5 Department of Mechanical Engineering, Northern Arizona University, Flagstaff, Arizona, United States of America; Università degli Studi di Milano: Universita degli Studi di Milano, ITALY

## Abstract

**Background:**

There is growing interest in the use of biofeedback-augmented gait training in cerebral palsy (CP). Audiovisual, sensorimotor, and immersive biofeedback paradigms are commonly used to elicit short-term gait improvements; however, outcomes remain variable. Because biofeedback training requires that individuals have the capacity to both *adapt* their gait in response to feedback and *retain* improvements across sessions, changes in either capacity may affect outcomes. Yet, neither has been explored extensively in CP.

**Methods:**

In this study, we evaluated the extent to which adolescents with CP (7M/1F; 14 years (12.5,15.26)) could adapt gait and retain improvements across four, 20-minute sessions using combined audiovisual and sensorimotor biofeedback. Both systems were designed to target plantarflexor activity. Audiovisual biofeedback displayed real-time soleus activity and sensorimotor biofeedback was provided using a bilateral resistive ankle exoskeleton. We quantified the time-course of change in muscle activity within and across sessions and overground walking function before and after the four sessions.

**Results:**

All individuals were able to significantly increase soleus activity from baseline using multimodal biofeedback (p < 0.031) but demonstrated heterogeneous adaptation strategies. In-session soleus adaptation had a moderate positive correlation with short-term retention of the adapted gait patterns (0.40 ≤ ρ ≤ 0.81), but generally weak correlations with baseline walking function (GMFCS Level) and motor control complexity (ρ ≤ 0.43). The latter indicates that adaptation capacity may be a critical and unique metric underlying response to biofeedback. Notably, in-session gains did not correspond to significant improvements in overground walking function (p > 0.11).

**Conclusions:**

This work suggests that individuals with CP have the capacity to adapt their gait using biofeedback, but responses are highly variable. Characterizing the factors driving adaptation to biofeedback may be a promising avenue to understand the heterogeneity of existing biofeedback training outcomes and inform future system optimization for integration into clinical care.

## Introduction

Among individuals with cerebral palsy (CP), over 70% walk independently or with a mobility aid [1], yet walking is often difficult and may degrade over time, due to a cascade of progressive musculoskeletal impairments (*e*.*g*., muscle weakness, contracture, and bony deformity) [2, 3]. Because mobility promotes independence in activities of daily living [4–7] and may slow the progression of musculoskeletal impairments [7–10], gait has become a key target in CP rehabilitation. A variety of intervention strategies have been developed to this end, including strength training, pharmaceutical prescriptions, and orthopedic and neurosurgeries [11]. It is not uncommon for an individual with CP to receive a combination of these interventions to improve and maintain mobility across the lifespan. However, in an effort to augment the current standards of care, there has been growing interest in using biofeedback-augmented gait training, as it facilitates task-specific and self-initiated walking practice, both of which may be critical for promoting neuroplastic changes [7, 12–17].

Biofeedback systems are commonly designed to provide audio, visual, or sensorimotor (*i*.*e*., tactile or proprioceptive) cues to reinforce desired gait patterns [18]. In CP, both audiovisual and sensorimotor systems have been used to elicit changes in gait speed [19–22], joint kinematics [19, 23, 24], muscle activity [19, 25], and, more recently, motor control [26]. Although these studies point to the potential efficacy of biofeedback training in CP, outcomes remain variable. For example, a recent study demonstrated that over 30% of the individuals with CP evaluated were not able to significantly modify ankle power, knee extension, or step length to reach established targets during visual biofeedback training [27]. Similarly, other studies have cited high interparticipant variability in speed, cost of transport, and functional gait outcomes for adults with CP following multi-session training with visual and proprioceptive biofeedback [22]. As such, before biofeedback can be a viable non-invasive rehabilitation strategy in CP, there is a need to better understand this heterogeneity in training responses.

Eliciting positive outcomes to biofeedback requires that individuals are able to both adapt gait and retain in-session improvements. This implies that deficits in either ability following neurologic injury may adversely affect response. Adaptation capacity during walking is commonly evaluated by systematically introducing a perturbation and measuring the time-course of changes in gait. Such perturbations have previously included adding weight to a leg or using a split-belt treadmill to drive both legs at different speeds [28–30]. These studies have reported that adaptation is commonly characterized by: (1) a gradual change in gait from baseline, as the central nervous system modifies its feedforward strategy through trial-and-error in response to the novel perturbation and (2) a brief persistence of the adapted gait pattern once the perturbation is removed, often called an aftereffect [31]. Studies have also noted that adaptation occurs more quickly with repeated practice, which is used to suggest that the central nervous system has partially retained the novel gait strategy to better accommodate the perturbation upon subsequent exposure [32]. As each of these processes involve supraspinal motor centers, adaptation capacity is often affected among those with neurologic injury and may even vary within subpopulations depending on the size and location of the primary injury [28]. For example, comparative studies in CP [29, 33] and stroke [34–38] have demonstrated that cerebral injury slows the rate at which individuals adapt to perturbation but does not significantly alter the presence of aftereffects. Further, recent work has shown that individuals with CP demonstrate faster adaptation across multiple sessions of split-belt treadmill walking, indicating that the central nervous system can still retain knowledge of novel perturbation environments following cortical injury [32].

Characterizing how individuals adapt gait in response to biofeedback may be critical to understanding the variability of outcomes. However, while these foundational studies in motor adaptation provide a valuable framework to understand adaptation capacity in CP, there is no evidence to suggest that the reported findings extend to rehabilitation. In fact, prior work has suggested that because adaptation is driven by error recognition, the extent to which individuals adapt their gait is likely contingent on both the type of perturbation and the way it is applied [29, 39]. As such, to understand outcome variability following biofeedback training, there is a need to directly evaluate adaptation within this context.

The aim of this study was to evaluate the extent to which individuals with CP adapt gait and retain improvements during multi-session practice with a multimodal biofeedback paradigm, designed to promote plantarflexor recruitment. Secondarily, we compared overground walking performance before and after biofeedback sessions to understand if any observed in-session improvements were transferred. We hypothesized that, similar to traditional adaptation studies, individuals would modulate plantarflexor activity in response to biofeedback and demonstrate aftereffects once the biofeedback was removed, suggesting that in-session gains were temporarily stored. We further hypothesized that the rate and magnitude of soleus adaptation would increase across sessions, indicating that individuals had retained knowledge of how to effectively engage with the biofeedback environment. Extending established frameworks in motor adaptation to evaluate biofeedback will bolster foundational understanding of adaptation capacity in CP and simultaneously provide insight into potential mechanisms which may underlie existing responses to biofeedback.

## Methods

### Participants

A convenience sample of eight adolescents with spastic cerebral palsy were recruited to evaluate adaptation to multimodal biofeedback (Table 1). To be eligible for participation, individuals had to be at least ten years old and be able to (1) walk for ten consecutive minutes on a treadmill, with handrails as needed, (2) follow simple verbal instructions, and (3) respond to audio and visual cues. The defined age cut-off was set to support task comprehension, as our prior research has highlighted that younger children often struggle to engage with multimodal biofeedback systems. Individuals were excluded from the study if they had received orthopedic surgery or lower limb botulinum toxin injections within the past six months, unless their participation was approved by their primary care provider and recent interventions were deemed to have limited impact on gait. Prior to data collection, all participants and their caregivers gave written consent and assent, as applicable, and the study protocol was approved by the Institutional Review Board at Northern Arizona University (#986744–28). All recruitment was conducted from June 1^st^–September 30^th^ of 2021.

**Table 1:** Participant demographics.

	Gender	GMFCS Level^a^	Diagnosis^b^	Age (yrs)	Height (m)	Mass (kg)	More-Affected Limb^c^	Walking Speed (ND)^d^
**P1**	M	II	SD	18	1.76	62.14	R	0.31
**P2**	F	II	SD	12	1.45	42.41	L	0.28
**P3**	M	III	SD	13	1.58	42.18	R	0.22
**P4**	M	II	SD	15	1.65	60.78	L	0.25
**P5**	M	II	SD	13	1.50	39.01	R	0.31
**P6**	M	I	SH	12	1.43	39.46	R	0.32
**P7**	M	III	SD	15	1.56	68.95	L	0.23
**P8**	M	I	SH	16	1.65	65.77	R	0.27

^a^Gross Motor Function Classification System; Level determined by licensed physical therapist

^b^Diagnoses include: Spastic diplegia (SD), spastic hemiplegia (SH); Diagnosis reported by caregivers

^c^Reported by participants

^d^Nondimensional, calculated according to Hof A.L., 1996

### Experimental protocol

**Fig 1:**
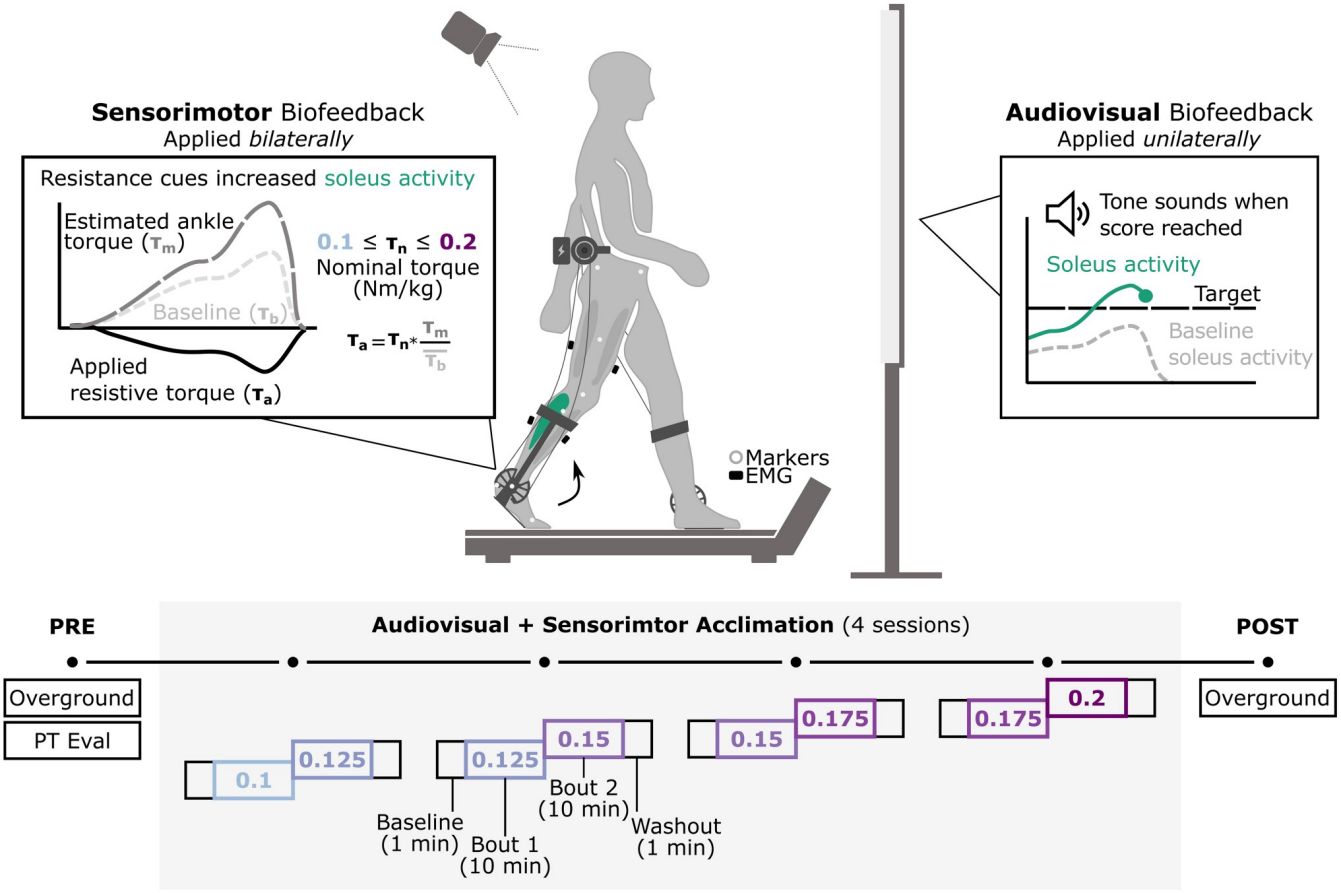
Experimental protocol used to evaluate multi-session adaptation to multimodal biofeedback. Participants completed a four-day protocol using combined audiovisual and sensorimotor biofeedback. Audiovisual biofeedback on soleus activity was provided unilaterally on the more-affected limb whereas sensorimotor biofeedback was administered bilaterally using a resistive ankle exoskeleton. Each session was separated into baseline (1 minute), biofeedback (2, 10-minute bouts), and washout (1 minute) phases. The nominal torque value of the ankle exoskeleton was set at 0.1 Nm/kg during the first bout of the first session and incrementally adjusted by 0.025 Nm/kg over the subsequent bouts, according to the schedule shown. Overground walking data were collected pre- and post-intervention. A licensed physical therapist also performed a full physical examination at the pre-intervention session. Motion capture data were collected during at the pre- and post-intervention sessions and electromyography (EMG) data were collected bilaterally from the vastus lateralis, semitendinosus, soleus, and tibialis anterior across all sessions.

Participants completed a four-day protocol during which they walked on a treadmill while responding to a multimodal biofeedback system (Fig 1). Each session was structured to include baseline walking (1 minute) immediately followed by biofeedback (two, 10-minute bouts) and washout (1 minute) phases. At the transition from the biofeedback to washout phases, the biofeedback system was automatically turned off and participants were instructed to walk in whatever pattern felt natural; this was included to capture aftereffects which could indicate that the central nervous system had temporarily retained in-session gait adaptations [31]. Between the two bouts of biofeedback walking during each session, participants were required to take a five-minute seated break to mitigate fatigue. Across all sessions, participants walked at a consistent self-selected speed (nondimensional speed; median (IQR): 0.27 (0.24,0.31)) and held onto treadmill handrails for safety.

Before and after the biofeedback protocol, participants completed assessments to measure if walking function significantly changed following intervention. During the pre-intervention session, participants were evaluated by a licensed physical therapist who confirmed their GMFCS level and more-affected limb and measured range of motion and spasticity at the knee (flexion/extension) and ankle (dorsi/plantarflexion). Participants then completed six overground barefoot walking passes at a self-selected speed. At the post-intervention session, only overground walking passes were collected. The full protocol was performed over a two-week period, with 1–3 days of separation between individual sessions.

### Multimodal biofeedback

At each biofeedback session, participants walked with audiovisual and sensorimotor biofeedback systems, both of which were designed to promote plantarflexor engagement (Fig 1). The plantarflexors were selected as they are critical for generating propulsive force during walking and are consistently affected in CP [3, 40]. Audiovisual biofeedback was administered *unilaterally* on the more-affected limb using a custom-built system (See [41] for full system details). Briefly, this system is designed to present the user with a real-time measure of soleus activity alongside a target score and play a tone any time that score is reached. Based on user success rate, the target score automatically adjusts, which helps to balance user engagement and task-challenge [42]. Sensorimotor biofeedback was administered *bilaterally*, using an untethered ankle exoskeleton that was developed for use in CP [43, 44]. This system is designed to impart a *resistive* (*i*.*e*., dorsiflexion) moment at the ankle proportional to the biological plantarflexion moment during walking to encourage greater plantarflexor recruitment. A custom-built MATLAB (MathWorks, Natick, USA) interface communicates with the device, allowing the researcher to adjust the nominal torque applied at the ankle within the session. Across biofeedback sessions, the nominal torque was adjusted from 0.1 Nm/kg to 0.2 Nm/kg in increments of 0.025 Nm/kg, normalized to participant bodyweight, according to a predetermined schedule (Fig 1); this schedule was selected based on prior training studies administered with the same system [26, 45]. During both the baseline and washout phases for each session, the audiovisual system was turned off and the exoskeleton was set to 0 Nm/kg nominal torque, which functionally turned both ankle assemblies into hinge joints.

It is important to note the discrepancy in how both modes of biofeedback systems were administered. While we were able to easily provide bilateral sensorimotor biofeedback, we elected to apply audiovisual biofeedback unilaterally, as we felt that bilateral audiovisual information may adversely affect user comprehension and subsequent outcomes. Although this introduced the potential for asymmetric gait effects, it also provided the opportunity to compare how response differed between a multimodal (*i*.*e*., audiovisual + sensorimotor) and unimodal (sensorimotor) biofeedback system [41].

### Data analysis

Bilateral surface electromyography (EMG) data were recorded for the tibialis anterior (TA), medial gastrocnemius (GAS), vastus lateralis (VL), and semitendinosus (ST) across all sessions (Noraxon; Scottsdale, AZ; 1000 Hz). All sensors were placed according to SENIAM guidelines. For each session, EMG data were high-pass filtered (40 Hz; 4^th^ order Butterworth), rectified, low-pass filtered (10 Hz; 4^th^ order Butterworth) and normalized to the 95^th^ percentile of the within-session baseline walking phase to generate linear envelopes.

Additionally, during the pre- and post-intervention sessions, synchronous motion capture data were collected using the Vicon Plug-In-Gait marker set (100 Hz; Fig 1) from which three-dimensional lower limb kinematics (Vicon Plug-In Gait Dynamic pipeline; Denver, CO) and spatiotemporal parameters (Gait Cycle Parameter Calculator; Vicon ProCalc) were derived [46, 47]. All overground data were segmented into individual strides, using the heel marker to delineate consecutive heel strikes, and time-normalized to 100 points.

#### Multi-session adaptation to biofeedback

For each biofeedback session, EMG data were separated into one-minute segments from which both peak muscle activity and motor control complexity were calculated. The latter was defined as the total variance that could be accounted for by a one-synergy solution (tVAF_1_), derived from non-negative matrix factorization [48]:

$$EMG_{m*t}=W_{m*1}*C_{1*t}+Error$$

$$tVAF_{1}=\left(1-\frac{Error^{2}}{EMG_{m*t}^{2}}\right)*100$$

Here, *m* is the number of muscles (*m* = 4), *t* is the number of time points, and *W* and *C* represent the synergy weights and their corresponding activation patterns, respectively. The tVAF_1_ is frequently used as a measure of motor control complexity because it is sensitive to walking function; individuals with CP and other neuromuscular conditions have higher tVAF_1_ values during walking than nondisabled peers which suggests that their motor control strategy is better-captured by a low-dimensional subspace and, therefore, less complex [49–52]. We hypothesized that across successive bouts, we would see an increase in peak soleus activity and decrease in tVAF_1_ (i.e., an increase in motor control complexity) which would indicate that individuals were significantly improving their motor control in response to biofeedback.

#### pre- and post-intervention analysis

To get a comprehensive understanding of whether in-session improvements using biofeedback were transferred to overground walking, we compared pre- and post-intervention data on multiple functional levels. Mean soleus activity across all overground strides was calculated to evaluate if muscle recruitment improved following multi-session practice with biofeedback. Further, joint-level changes in gait were evaluated using the gait deviation index (GDI). GDI is a summary measure of kinematics that is used to quantify the extent to which three-dimensional pelvis, hip, knee, and ankle angles align with nondisabled trends [53]. GDI scores are scaled such that a score of 100 indicates the average gait pattern for nondisabled individuals and lower numbers indicate increasing levels of gait impairment. For each recorded stride, the GDI was computed using a previously published nondisabled data set [53]. Finally, changes in full-body coordination were evaluated using spatiotemporal parameters (*i*.*e*., speed, step width, step length, cadence) and motor control complexity (tVAF_1_). The tVAF_1_ was quantified using EMG data for seven strides, concatenated across all overground walking passes for each session; the number of strides was held constant for all participants and sessions because tVAF_1_ is sensitive to the amount of variance in the input data [54]. For any participants that had more than seven recorded strides, strides were randomly sampled without replacement (replicates = 50) to generate a distribution of tVAF_1_ values from which the mean value was then used.

As a secondary analysis, we compared whether individual differences in walking function, defined by GMFCS level and motor control complexity (tVAF_1_) during the pre-intervention session were correlated with in-session gait adaptation and aftereffects. We hypothesized that individuals with better baseline walking function would have the largest change in gait while using biofeedback and the largest aftereffects.

### Statistical analysis

For all biofeedback sessions, we evaluated changes in peak soleus activity and motor control complexity (tVAF_1_) from baseline at three phases within each bout—early (first minute), mid (minutes 3–8) and late adaptation (last minute)–as well during washout. We used multiple Wilcoxon signed-rank tests and adjusted for multiple comparisons using a Holm-Šídák correction. Further, multiple Friedman tests were used to compare response in each phase (*i*.*e*., early, mid, and late adaptation) across bouts; for those tests which reached significance, post-hoc pairwise comparison was performed using Wilcoxon signed-rank tests. Spearman rank correlation coefficients (ρ) were used to quantify the association between soleus activity and the activity of the other muscle (*i*.*e*., TA, ST, VL), to understand if individuals were able to selectively modify control or displayed multi-muscle adaptations in response to biofeedback. Spearman rank correlation coefficients were also used to understand if response during biofeedback walking was associated with (1) response during washout and (2) walking function, as measured using participants’ GMFCS levels and baseline tVAF_1_. Finally, overground walking function (*i*.*e*., muscle activity, GDI, and tVAF_1_) before and after the biofeedback sessions were compared using Wilcoxon signed-rank tests. All results are presented as median (IQR) values unless otherwise noted. Deidentified data for all analyses are provided as Supporting Information (S1 File). All analyses were performed using the MATLAB Statistical Toolbox (MathWorks, Natick, USA) and significance was defined as p < α for α = 0.05.

## Results

**Fig 2:**
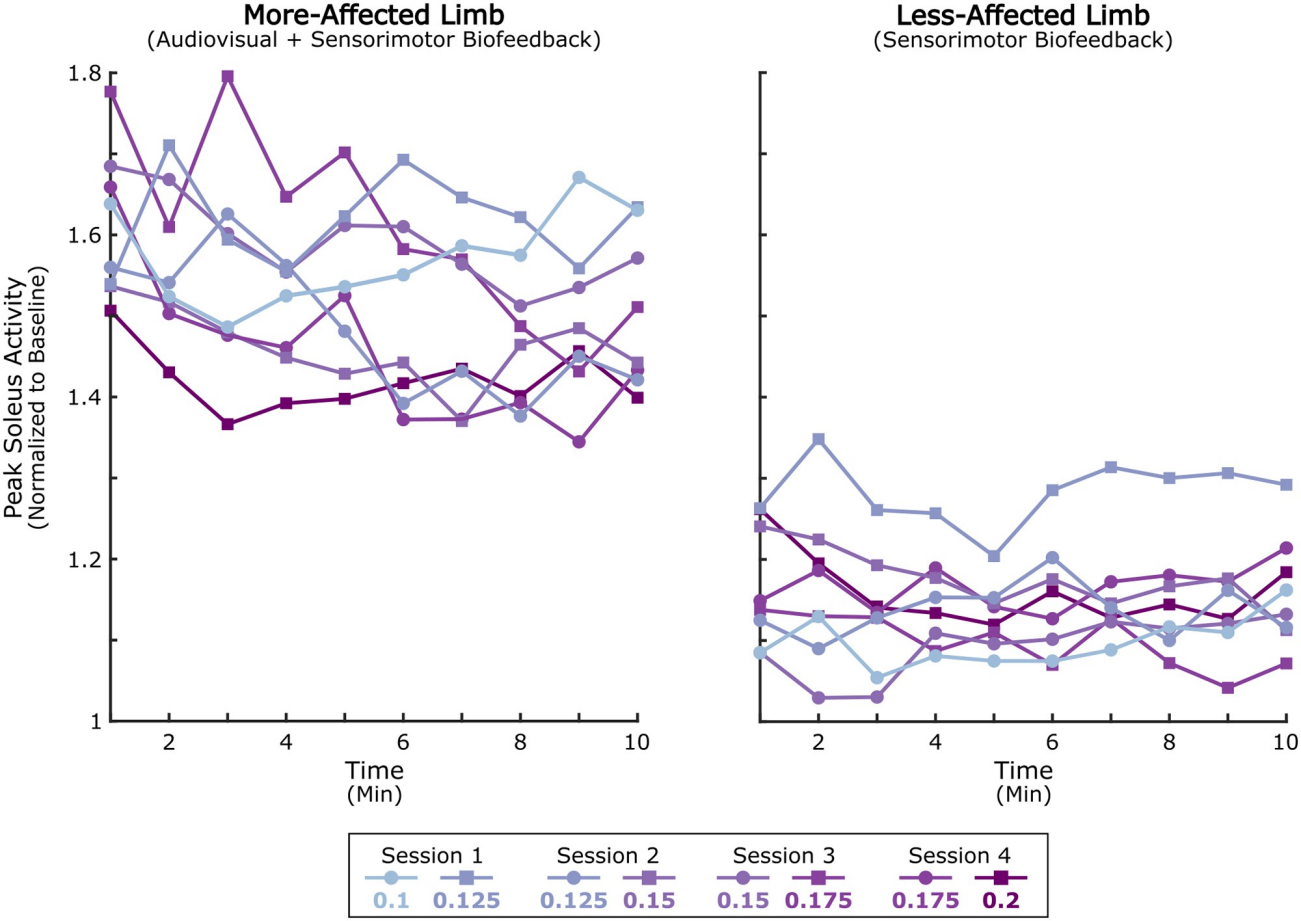
In-session soleus adaptation to multimodal biofeedback. Median trend in peak soleus activity on the more-affected limb (left) and less-affected limb (right) for each minute of every ten-minute bout. Participants performed two 10-minute bouts in every session (1–4). The nominal resistance level applied by the sensorimotor biofeedback system was incrementally increased from 0.1 to 0.2 Nm/kg (normalized to participant bodyweight), according to the legend, where the left and right values under each session heading correspond to Bout 1 and 2, respectively. Soleus activity for each session was normalized to the 95^th^ percentile of the in-session baseline walking phase. Both audiovisual (AV) and sensorimotor (SM) biofeedback were administered on the more-affected limb, whereas only SM biofeedback was administered on the less-affected limb so as to not impact user comprehension.

The rate at which individuals adapted to biofeedback did not change across sessions. Within the first minute of the biofeedback systems being turned on in each bout (i.e., early adaptation), participants increased peak soleus activity from baseline values (Fig 2). However, there was no difference in response between all sessions or bouts (Friedman; p = 0.53), indicating limited effect of the SM resistance level or additional practice on the rate of adaptation. Interestingly, this initial response to biofeedback was transient in-session; for the majority of bouts, there was a moderate negative correlation between peak soleus activity and time (Spearman Rank Correlation: -0.86 ≤ ρ ≤ -0.52 for all but Session 1 Bout 2 and Session 4 Bout 2) (Fig 2). This was most notable in the final biofeedback session, as soleus activity decreased by 48.0 (25.4, 61.2) percentage points and 25.1 (12.6, 75.1) percentage points on average between early (i.e., first minute) and late (i.e., last minute) adaptation for Session 4 Bout 1 and Bout 2, respectively. The attenuation observed throughout each biofeedback bout is a marked deviation from traditional adaptation paradigms which often report a plateauing of response and suggests that participants, despite adapting quickly to biofeedback, could not reliably maintain their initial level of engagement [32, 55]. Given this trend, we elected to use minutes 3–8 of the feedback trial (i.e., mid adaptation) for all remaining analyses.

**Fig 3:**
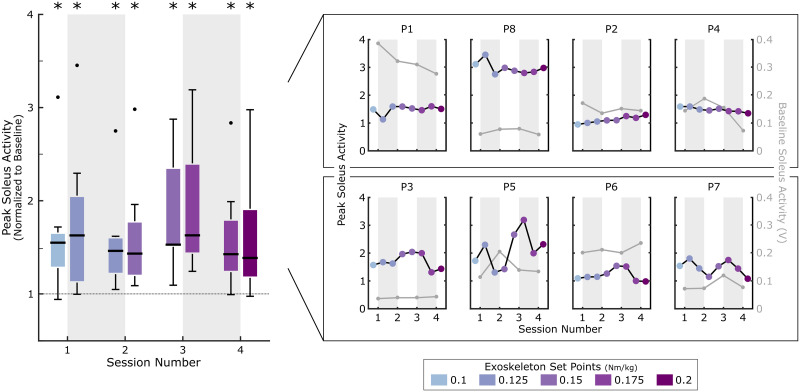
Peak soleus activity across four sessions with multimodal biofeedback. Peak soleus activity for the more-affected limb across all sessions with combined audiovisual and sensorimotor biofeedback. Each session was separated into two 10-minute bouts of biofeedback walking. Data represent activity from minutes 3–8 of each bout, defined as the mid-adaptation response. For each session, data were normalized to the 95^th^ percentile of the one-minute baseline phase. A resistive ankle exoskeleton was used to provide sensorimotor biofeedback and the nominal resistance level was incrementally increased from 0.1 Nm/kg to 0.2 Nm/kg (normalized to participant bodyweight) across sessions, according to the figure legend. The left panel depicts group-wise trends, and the right panel shows individual responses as well as baseline soleus activity values (used for normalization).*indicates a significant change in soleus activity from baseline.

At mid-adaptation, participants increased peak soleus activity on their more-affected side by between 39.2% (18.7, 91.2) (Session 4, Bout 2) to 63.5% (44.4, 139.8) (Session 3, Bout 2) above baseline (p < 0.031 for all bouts; Fig 3). However, consistent with the observations in adaptation rate, there was no significant difference in response between bouts (Friedman; p = 0.35). Despite these group-wise trends, there was a notable amount of variability in response at the individual level (Fig 3); some participants had the greatest response in Session Three with a sharp decline in Session Four (P3, P5, P6) whereas others (P1, P8, P4) had generally consistent changes in soleus activity across all sessions, even though the nominal resistance applied by the sensorimotor biofeedback system was incrementally increased. Only one participant (P2) demonstrated the expected monotonic increase in soleus activity across bouts. Interestingly, baseline motor control complexity (tVAF_1_; ρ ≤ 0.43) and GMFCS (ρ ≤ 0.23) had generally weak correlations with soleus modulation during biofeedback walking across sessions, indicating that factors beyond baseline functional ability may underlie individual response to biofeedback.

On the less-affected limb, where sensorimotor feedback but not audiovisual feedback was being provided, response was limited. The median increase in soleus activity from baseline was between 8.1% (-.01,24.4) (Session 1 Bout 1) and 27.1% (0,65.6) (Session 1 Bout 2), with only one bout reaching significance at the group level (S1 Fig; p = 0.046 for Session 3, Bout 1). This discrepancy highlights the advantage of augmenting sensorimotor paradigms with audiovisual feedback to focus attention and enhance user response [41].

**Fig 4:**
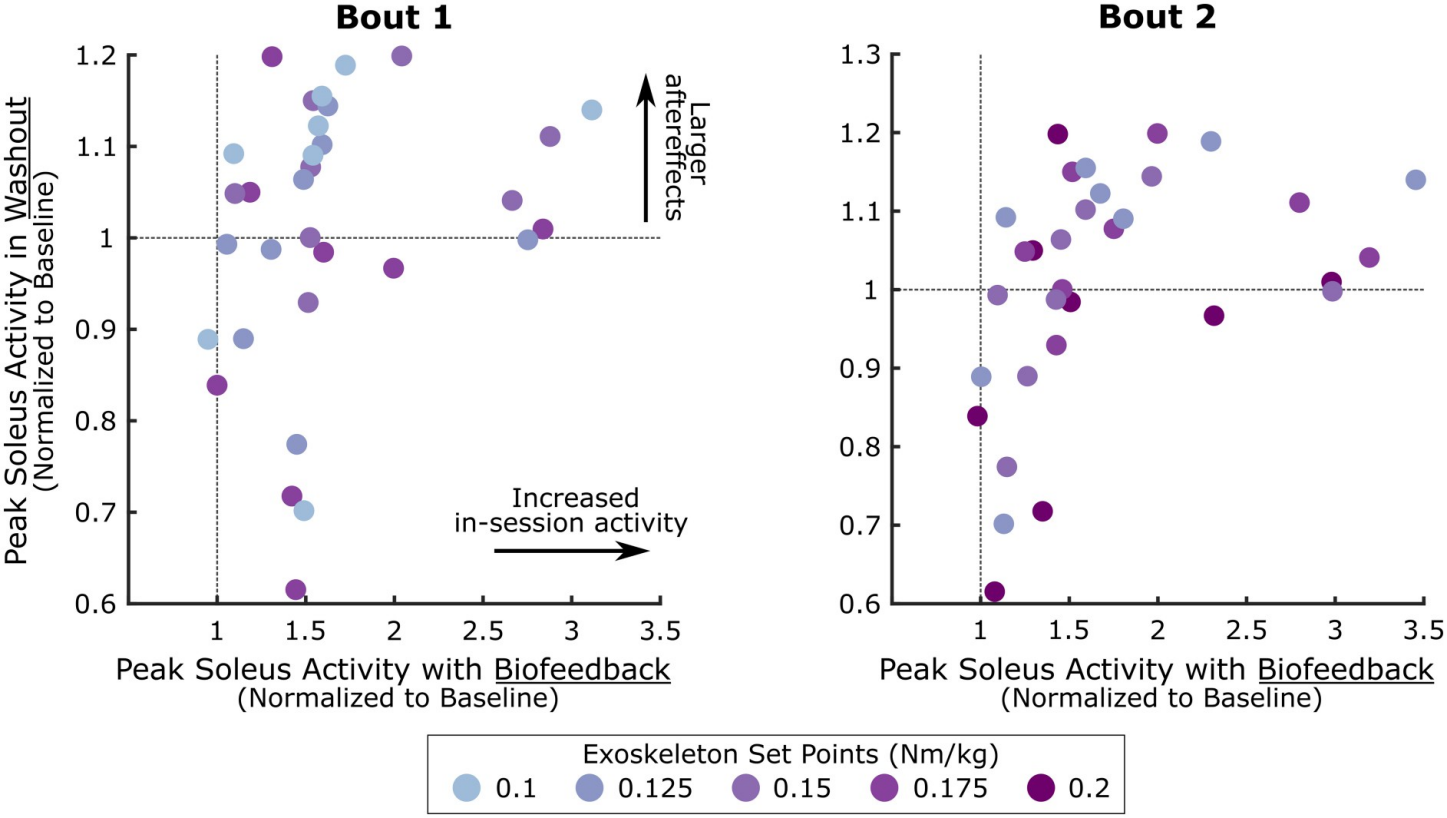
Correlation between peak soleus activity during biofeedback and washout phases. Mid-adaptation (minutes 3–8) peak soleus activity for each participant’s (n = 8) more-affected limb during the first (left) and second (right) bout of each biofeedback session compared to the washout phase. The washout phase represents the time at which biofeedback was turned off, which was used to evaluate short-term retention of in-session gains. All data have been normalized to the 95^th^ percentile of baseline activity for each session. Individual dots represent participants and colors indicate the magnitude of the resistance applied bilaterally by the ankle exoskeleton (i.e., sensorimotor biofeedback), normalized to participant bodyweight.

Once biofeedback was removed (*i*.*e*., washout), the presence of aftereffects was related to the in-session response (Fig 4). There was a moderate to strong positive correlation between the magnitude of soleus activity mid-adaptation and during washout for all bouts (0.40 ≤ ρ ≤ 0.81), except for the first bout of Session 4 (ρ = -0.05). Interestingly, this relationship appeared nonlinear, whereby those who modified soleus activity to the greatest extent had aftereffects of similar magnitude to those who had more moderate changes in-session. This suggests that there may be a threshold on the extent that an adapted gait pattern is retained immediately following removal of biofeedback.

**Fig 5:**
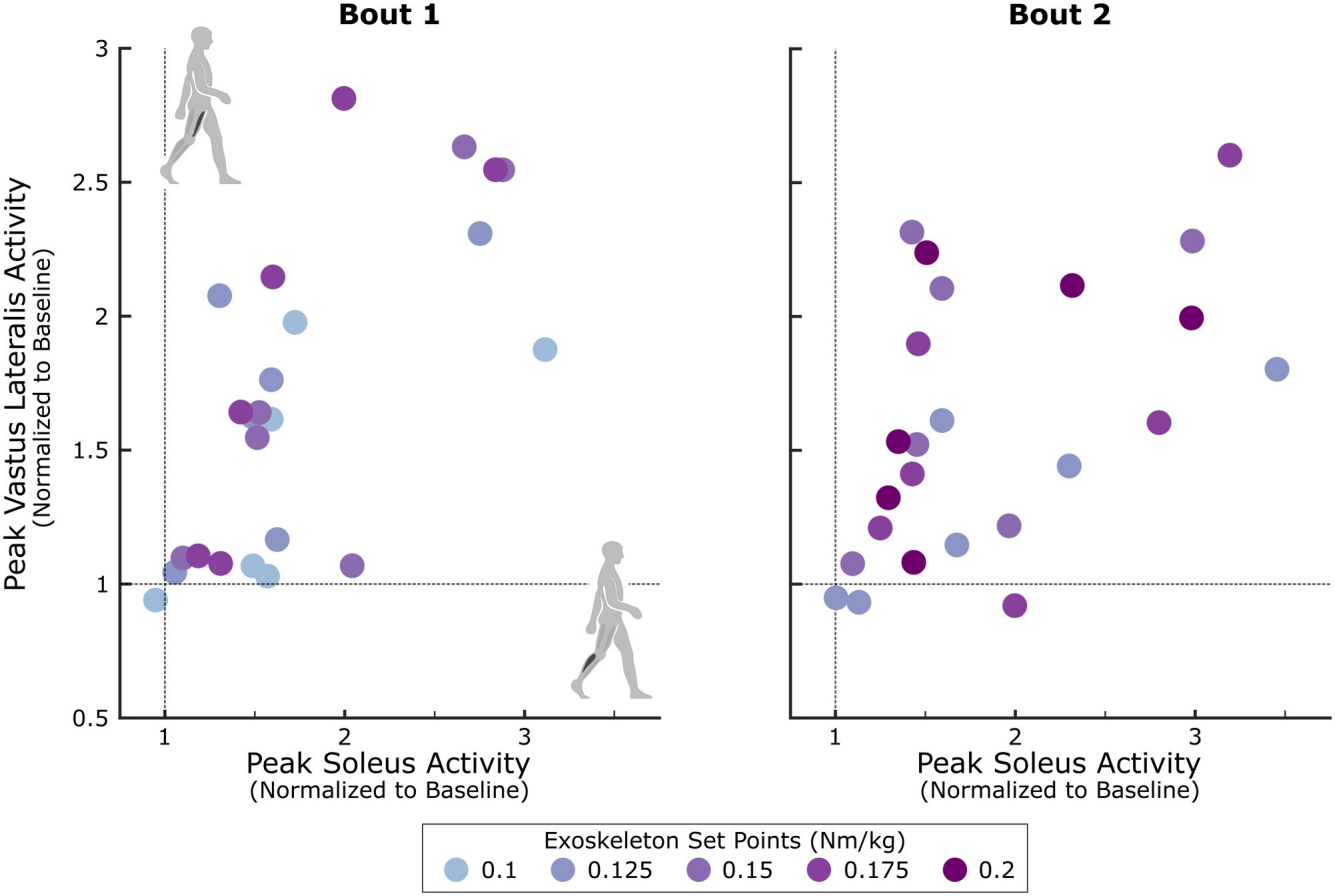
Correlation between peak soleus activity and peak vastus lateralis activity across biofeedback sessions. Mid-adaptation (minutes 3–8) peak soleus activity for each participant’s more-affected limb compared with their peak vastus lateralis activity. Data has been normalized to the 95^th^ percentile of baseline walking and separated into the first (left) and second (right) bouts for each session. Individual dots represent participants and colors indicate the magnitude of the resistance applied bilaterally by the ankle exoskeleton (i.e., sensorimotor biofeedback), normalized to participant bodyweight. Note that these trends represent six of the eight participants included in this study, as two (P6 and P7) had to be removed due to EMG signal loss.

In parallel with the changes observed in soleus recruitment during biofeedback walking, there were simultaneous changes in proximal muscle activity. In general, participants tended to adopt less complex motor control strategies in response to biofeedback, characterized by increased co-contraction of antagonist muscle groups. Across sessions, peak vastus lateralis activity increased by between 29.4% (-5.1,61.1) (Session 1, Bout 2) and 89.4% (10.5, 154.7) (Session 4, Bout 1) above baseline while peak semitendinosus activity increased from baseline by between 16.1% (-1.8, 36.5) (Session 2, Bout 2) and 38.1% (6.0, 54.6) (Session 4, Bout 1), although none of these group-wise changes reached significance (p > 0.062). Further, there was a moderate to strong positive correlation (0.48 ≤ ρ ≤ 0.88 for all but Session 2 Bout 2) between vastus lateralis and soleus activity (Fig 5). In line with observed changes in proximal muscle activity, motor control complexity (tVAF_1_) increased between 4.0% (1.3,7.4) (Session 4, Bout 1) and 8.8% (6.0,15.3) (Session 3, Bout 2) above baseline across bouts, reaching significance for the first bout of Sessions 2 and 3 (p = 0.03). Note that all multi-muscle analysis was performed on a subset of the population (n = 6), due to EMG signal losses for the vastus lateralis for P6 and P7.

**Fig 6:**
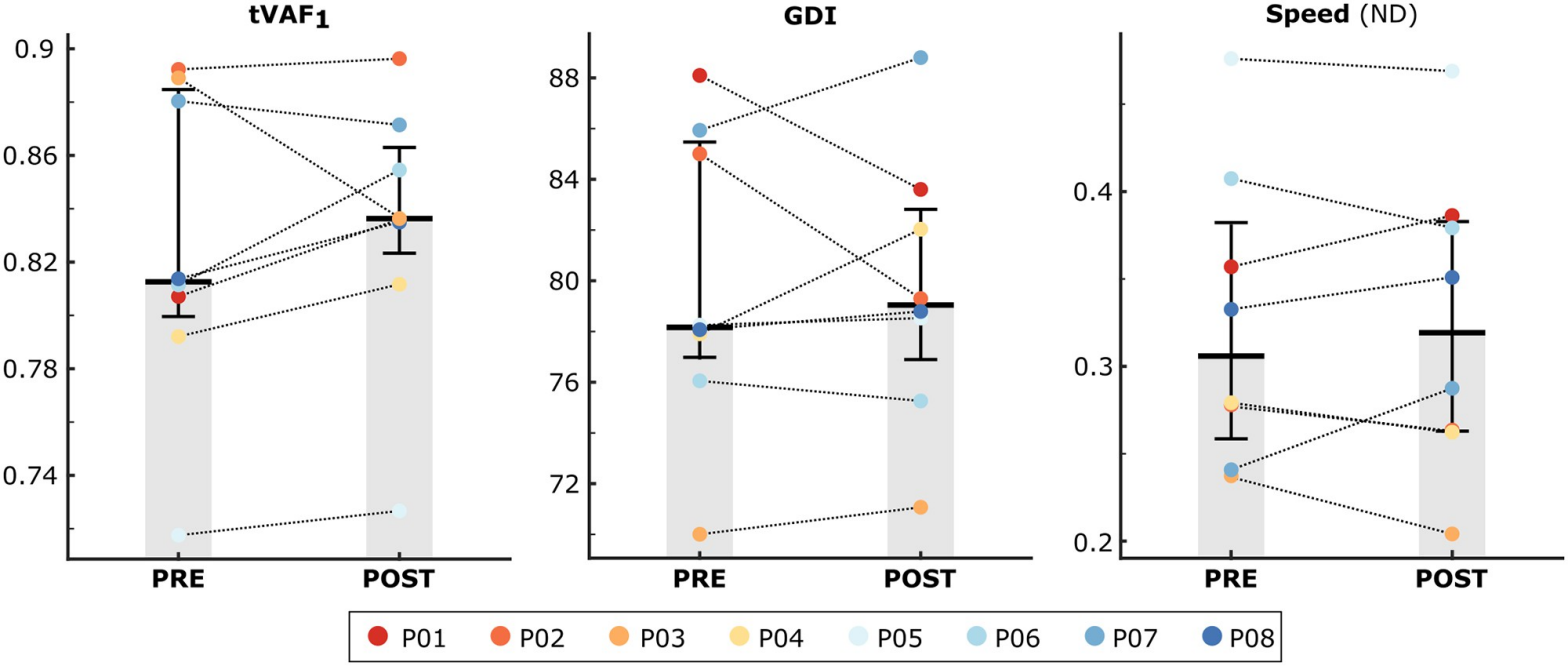
Functional measures of overground walking before and after multi-session adaptation to biofeedback. Motor control complexity (tVAF_1_), gait deviation index (GDI), and walking speed (non-dimensional) were measured during overground walking before and after the biofeedback sessions. Bar plots represent the median (IQR) value across individuals and colored points represent participant data. No significant changes in walking function were observed following multi-session adaptation to combined audiovisual and sensorimotor biofeedback.

Despite the observed in-session changes in soleus recruitment across all bouts, gains were largely context-specific, as there were no significant changes in overground walking function at the muscle, joint, or full-body level follow multi-session adaptation to biofeedback (Fig 6). Specifically, mean soleus activity during stance (Post-Pre Difference = 0.02 (-0.01,0.08) percentage points; p = 0.38), non-dimensional step width (0.01 (-0.02,0.01); p = 0.55), step length (-0.01 (-0.05,0); p = 0.11), and speed (-0.01 (-0.02,0.02); p = 1.0), motor control complexity (0.01 (0, 0.03); p = 0.31), and GDI (0.5 (-2.6, 2.0); p = 1) were similar during both the pre- and post-intervention sessions.

## Discussion

Using audiovisual and sensorimotor biofeedback, participants significantly modified soleus activity from baseline. However, counter to our original hypothesis, there was no consistent increase in either the rate or magnitude of soleus modulation across sessions. Instead, we noted a significant amount of interparticipant variability to biofeedback that was not directly correlated with participants’ baseline walking function. There was a notable positive correlation between in-session soleus modulation and aftereffects, whereby greater changes in soleus activity during biofeedback walking generally corresponded to larger aftereffects once the systems were turned off. However, these short-term adaptations were not transferred beyond the biofeedback sessions.

That a diverse cohort of adolescents with CP increased soleus activity in response to multimodal biofeedback indicates that such systems may be advantageous for pediatric gait rehabilitation. However, the heterogeneity of response observed between participants and across sessions warrants additional evaluation, as it may inform how candidates are identified and selected for biofeedback training and how biofeedback systems and training programs are developed to optimize outcomes.

Given the complex user-system interactions inherent in biofeedback training paradigms, there were likely many factors which likely influenced individuals’ response to multimodal biofeedback. In particular, differences in compensation strategies may have contributed to the interparticipant heterogeneity we observed. While both biofeedback systems were designed to directly target soleus activity, we noted a significant change in multi-muscle control, indicating that individuals were adopting full-limb compensatory strategies during biofeedback walking. Prior work has demonstrated that because the sensorimotor system used in this study actively resists plantarflexion, individuals may increase hip and knee flexion, and correspondingly proximal muscle activity, during walking to bypass the effects of the device [41, 56]. Pairing this system with an audiovisual system that provided real-time information on soleus activity may have promoted greater engagement with the sensorimotor system and, therefore, simultaneous increases in soleus and proximal muscle activity. However, the extent that individuals compensated or responded to both biofeedback systems likely changed as a function of the resistance level applied by the sensorimotor system and fatigue, contributing to the overall intersession and interparticipant variability observed. Collectively, this points to a shortcoming of the biofeedback system designs used in this study and highlights the need to closely monitor compensation or present multidimensional cues during biofeedback training to avoid potentially undesirable compensation.

Beyond compensation, differences in sensory integration may have also contributed to in-session response. The central nervous system relies on sensory information to detect errors between predicted and actual movement patterns and update its internal models (*i*.*e*., sensorimotor calibration) of the environment accordingly. As such, if these afferent pathways are disrupted due to neurologic damage, adaptation may occur more slowly or even incompletely [32]. This is supported by prior evidence which demonstrates that individuals with cerebral injury due to CP, stroke, traumatic brain injury, or hemispherectomy adapt more slowly to split-belt treadmill or single-leg weighting perturbations than nondisabled peers [28, 29, 33, 57, 58]. Further, recent work reported that there is a negative correlation between the variability of proprioceptive signals and visuomotor adaptation rates in older adults during reaching tasks [59]. While the intent of providing augmented audiovisual and sensorimotor biofeedback was to supersede these potentially unreliable sources of afferent information and, therefore, facilitate greater adaptation, discrepancies in sensory information may still have contributed to the observed heterogeneous response. As such, integrating measures of proprioception into biofeedback studies in CP may provide additional insight into the potential mechanisms underlying adaptation.

We also observed that in-session gains were positively correlated with aftereffects during washout. Prior work has hypothesized that the aftereffects observed once a perturbing environment has been removed provide insight into the extent to which the central nervous system has temporarily modified its internal model to meet the task demand [60]. Our findings demonstrated that those individuals with greater adaptation capacity generally had larger aftereffects; however, the relationship was nonlinear, as the magnitude of aftereffects appeared to plateau. Interestingly, we also found that baseline functional measures of gait (*i*.*e*., motor control complexity and GMFCS) were not strongly correlated with the magnitude of adaptation for any session (ρ ≤ 0.43). These findings align with prior work in individuals following hemispherectomy and stroke which reported that there was no correlation between the extent of adaptation and degrees of clinical impairment [34, 58, 61]. As the cerebellum, which is commonly unaffected in CP, is primarily thought to be involved in adaptive processes, this finding is not entirely unexpected [28]. However, it highlights that beyond standard functional measures, more direct measures of adaptation capacity may be critical to consider in clinical decision making when identifying candidates for biofeedback paradigms.

The biofeedback protocol may have also affected individual responses across sessions and outcomes during overground walking. In biofeedback system design there are myriad tunable parameters that may influence response including the modality used, the gait metric targeted, and the frequency and timing of individual cues [12]. In this study, we provided concurrent biofeedback across every session. While this may be advantageous in the early stages of learning a novel task, it can simultaneously foster reliance on extrinsic sources of error information over intrinsic pathways, such that once the system is removed, individuals return to baseline function [18, 62–64]. To counter this, prior evidence has demonstrated that providing feedback at the end of a task (often called a ‘knowledge of results’ paradigm) or interleaving randomized non-feedback cycles into sessions may strengthen individuals’ capacity to independently recognize and correct movement error [12, 18]. Biofeedback system design choices may also influence participant engagement, task comprehension, and focus; what might be interpretable, interesting, and motivational to some participants may be less so for others. In post-hoc analysis, we did note a moderate to strong positive correlation between participant age and adaptation to biofeedback (0.47 ≤ ρ ≤ 0.75 for all but Session 3). This suggests that the biofeedback systems as designed may have been more effective for the older participants and highlights the need for biofeedback systems to be intentionally designed based on the target audience. Administering biofeedback on a treadmill may have also attenuated transfer to overground walking. Not only did the treadmill protocol restrict individuals to walking at a consistent speed, which may be disadvantageous when attempting to modulate plantarflexor activity, but prior work has demonstrated that adaptation may be highly context dependent. Specifically, the visual-proprioceptive mismatch inherent in treadmill training paradigms has been hypothesized to cause individuals to develop environment-specific internal models, meaning that task learning may improve but may not be generalizable across all walking configurations [65]. Finally, the structure of the multi-session protocol, specifically the length of each session, number of sessions tested, and the time between individual sessions may have influenced both intersession response and transfer. Prior work demonstrated that a ten-session training protocol with the same sensorimotor biofeedback system used in this study was sufficient to elicit changes in both motor control and overground walking function in individuals with CP [26]. Further, a previous study demonstrated that individuals with CP continued to show improvements in adaptation to split-belt treadmill walking across thirty practice sessions [32]. These studies suggest that we may have provided insufficient practice for individuals to see measurable improvements in walking performance. Variable time between sessions may have had a similar effect. Although all sessions were spaced 1–3 days apart, this timing may have introduced distinct confounders; scheduling sessions too close together may have provided insufficient recovery time, and therefore increased the likelihood of rapid fatigue, whereas scheduling sessions too far apart may have resulted in prior gains being fully washed out.

While this study marks a critical step in understanding multi-session adaptation to biofeedback in CP, there are limitations that must be considered in interpreting the results. We reported that the rate of adaptation did not change across sessions by comparing data from the first minute of walking in each bout. This was motivated, in part, by prior studies which have demonstrated that adaptation to split-belt treadmill and single-leg weighting paradigms typically occurs over a 10–15 minute window in nondisabled adults and occurs even more slowly for those with neuromuscular impairments [55, 66–68]. However, it is possible that there may have been differences in response between bouts on a shorter time scale than we were able to measure [41]. Due to time constraints in our protocol and the limitations of our data collection space, we were unable to measure gait kinematics or kinetics during biofeedback sessions and therefore could not evaluate soleus adaptation on a stride-by-stride basis. This also restricted us from measuring how stride-to-stride variability changed within and across sessions, which may be a critical metric to characterize both learning and fatigue [32]. Together, these limitations highlight a critical next step of this work that will further advance understanding of how adaptation changes as a function of practice. Further, given the difficulty of collecting reliable maximum voluntary isometric contractions for individuals with CP [69], we elected to normalize EMG data to the peak baseline activity within each session. While there was no difference in baseline activity across sessions at the group level (Friedman; p = 0.58), thereby justifying our decision to make intersession comparisons, some participants did demonstrate deviations in baseline activity which could have influenced the observed trends (Fig 3). This highlights an inherent limitation of evaluating EMG data across multi-session protocols, as effective normalization parameters are challenging to establish, particularly among those with neuromuscular disorders. Given previous challenges that we have observed in engaging young children with both the exoskeleton and audiovisual systems used in this study, we also excluded children under ten years old. However, as evidence suggests that gait rehabilitation may be most effective in early childhood, future work should develop biofeedback paradigms that are better suited for younger children [70]. Finally, as this study was our first attempt to quantify multi-session adaptation, we intentionally selected a smaller sample size which, although similar to prior biofeedback and exoskeleton studies in CP, directly affects the generalizability of our results and our statistical power [26, 45]. Despite the small size, the heterogeneity of our cohort enabled us to capture many different responses to biofeedback and demonstrate the feasibility of using this paradigm for individuals across GMFCS Levels I-III. However, additional testing with a larger sample size is needed to more comprehensively evaluate adaptation capacity in CP as well as the range of factors that may dictate different responses to biofeedback; the latter is essential for refining future biofeedback protocols and system designs for rehabilitation.

## Conclusion

This study demonstrated that individuals with CP can significantly modify gait in response to combined audiovisual and sensorimotor biofeedback, but do not demonstrate consistent intersession improvements in the rate or magnitude of adaptation. However, it also revealed a high level of interparticipant variability in both adaptation capacity and aftereffects. Understanding the system and participant-level factors which influence response to biofeedback is a critical area for future research. Not only will this aid in the intentional design of biofeedback systems and training protocols but it will also help to identify individuals that may benefit from training, ultimately improving the efficacy of biofeedback as a clinical rehabilitation strategy.

## Supporting information

S1 File Deidentified project data. This compressed folder contains all participant-level data for pre-, post-, and training visits used for the presented analyses and figures. The file READ ME.txt contains basic definitions for all presented variables. All data has been de-identified to protect participant privacy.
(ZIP)

S1 Fig Peak soleus activity for the less-affected limb across four sessions with sensorimotor biofeedback. Peak soleus activity for the less-affected limb across all sessions with sensorimotor biofeedback. Each session was separated into two 10-minute bouts of biofeedback walking. Data represents activity from minutes 3–8 of each bout, defined as the mid-adaptation response. For each session, data were normalized to the 95^th^ percentile of the one-minute baseline phase. A resistive ankle exoskeleton was used to provide sensorimotor biofeedback and the nominal resistance level was incrementally increased from 0.1 Nm/g to 0.2 Nm/kg (normalized to participant bodyweight) across sessions, according to the figure legend. *indicates a significant change in soleus activity from baseline.
(TIF)
