# Supplementary figures and images for: Multi-session adaptation to audiovisual and sensorimotor biofeedback is heterogeneous among adolescents with cerebral palsy
Source: PLoS One. 2024 Nov 18;19(11):e0313617. doi: 10.1371/journal.pone.0313617 (PMC11573209; doi:10.1371/journal.pone.0313617)

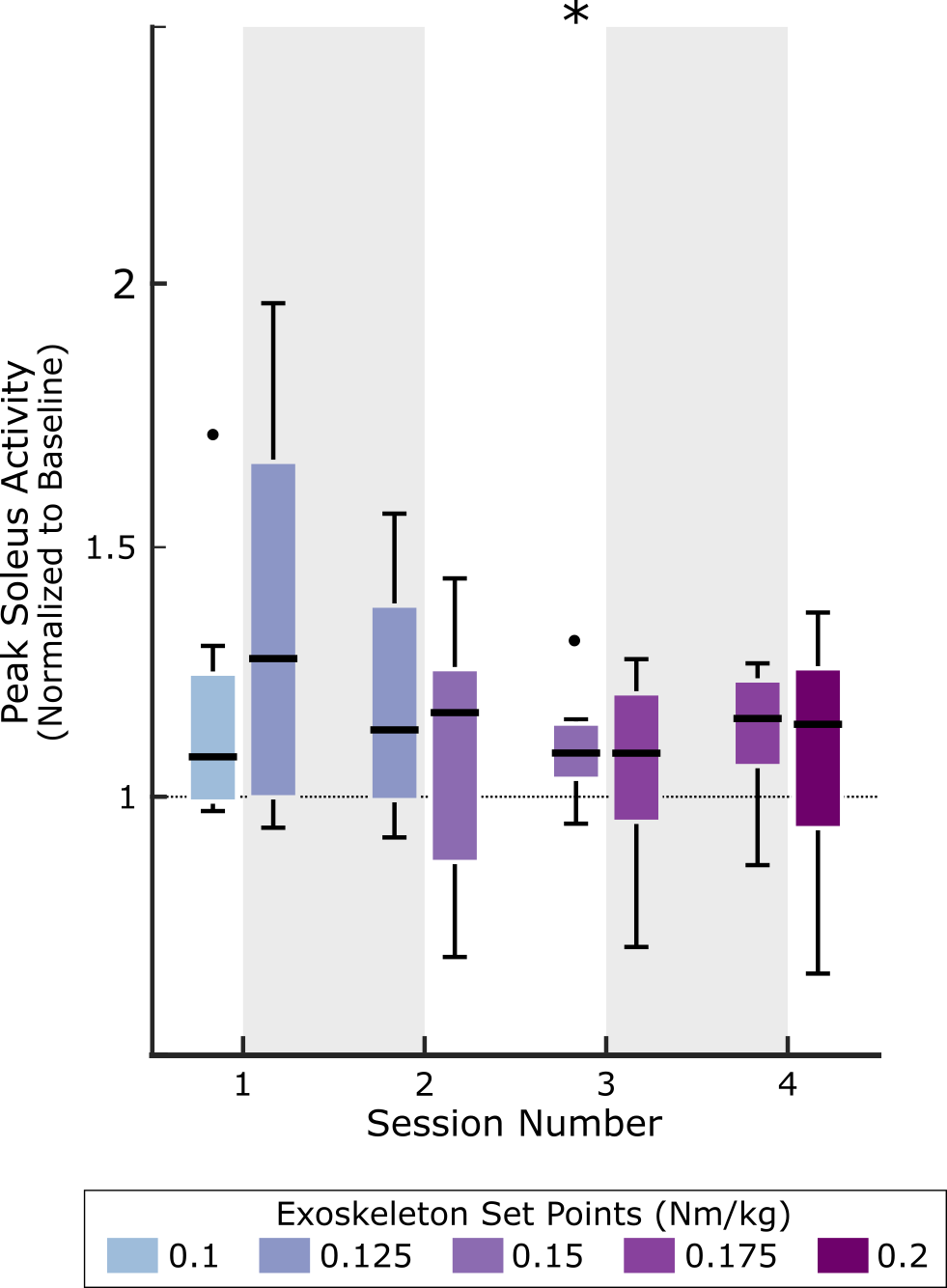

Supplement: S1 Fig — Peak soleus activity for the less-affected limb across all sessions with sensorimotor biofeedback. Each session was separated into two 10-minute bouts of biofeedback walking. Data represents activity from minutes 3–8 of each bout, defined as the mid-adaptation response. For each session, data were normalized to the 95th percentile of the one-minute baseline phase. A resistive ankle exoskeleton was used to provide sensorimotor biofeedback and the nominal resistance level was incrementally increased from 0.1 Nm/g to 0.2 Nm/kg (normalized to participant bodyweight) across sessions, according to the figure legend. *indicates a significant change in soleus activity from baseline. (TIF) [file pone.0313617.s002.tif]
